# Comparison of In Vitro and In Vivo Antioxidant Activities of Six Flavonoids with Similar Structures

**DOI:** 10.3390/antiox9080732

**Published:** 2020-08-11

**Authors:** Yixiu Zeng, Jiajia Song, Meimei Zhang, Hongwei Wang, Yu Zhang, Huayi Suo

**Affiliations:** College of Food Science, Southwest University, Chongqing 400715, China; chestnut@email.swu.edu.cn (Y.Z.); jiajias@swu.edu.cn (J.S.); zmm318725@email.swu.edu.cn (M.Z.); wanghw_1978@swu.edu.cn (H.W.); y000063@swu.edu.cn (Y.Z.)

**Keywords:** flavonoid, antioxidant, structure, d-galactose

## Abstract

The in vitro and in vivo antioxidant activities of six flavonoids with similar structures, including epicatechin (EC), epigallocatechin (EGC), procyanidin B2 (P), quercetin (Q), taxifolin (T), and rutin (R) were compared. The structures of the six flavonoids and their scavenging activities for 2,2-diphenyl-1-picrylhydrazyl (DPPH•) and 2,2′-azino-bis-(3-ethylbenzothiazoline-6-sulfonic acid) (ABTS^+^) radicals were closely related. The flavonoids decreased serum contents of malondialdehyde (MDA) and nitric oxide (NO), and increased serum total antioxidative capacity (T-AOC), superoxide dismutase (SOD), catalase (CAT), and glutathione peroxidase (GSH-Px) levels to different degrees in d-galactose-treated mice. The changes in mRNA expression of liver GSH-Px1, CAT, SOD1, and SOD2 by d-galactose were dissimilarly restored by the six flavonoids. Moreover, the six flavonoids differentially prevented the inflammatory response caused by oxidative stress by inhibiting interleukin (IL)-1β, IL-6, and tumor necrosis factor (TNF)-α levels, and restoring IL-10 levels. These six flavonoids from two subclasses revealed the following antioxidant capability: P > EC, EGC > EC, Q > T, Q > R. Our results indicate that (1) the pyrogallol, dimerization, and C2=C3 double bonds of flavonoids enhanced antioxidant activity and (2) the C3 glycosylation of flavonoids attenuated antioxidant capacity.

## 1. Introduction

The aging process is accompanied by complex pathological changes, and aging is strongly related to a decline in physical function and an increased risk of death [1]. Aging is considered the primary driver in the development of human disease, and is one of the most important risk elements of stroke, neurodegenerative diseases, diabetes, and cancer [1,2]. The core feature of aging-related diseases is oxidative stress, as oxidative stress produces large amounts of reactive oxygen species (ROS) [3,4,5]. Damage caused by oxidative stress can be delayed or prevented by antioxidants [6]. Therefore, seeking an effective antioxidant strategy is essential for anti-aging and treatment of aging-associated diseases.

The side-effects of synthetic antioxidants have become increasingly prominent [7]; therefore, antioxidants from plants have gained increasing importance. Flavonoids are plant secondary metabolites, which are a type of phenol with antioxidant capacity. The flavonoid extracted from mulberry fruit exhibits a strong antioxidant effect in vitro, and inhibits H_2_O_2_-induced hemolysis of mouse red blood cells and lipid peroxidation in the liver, mitochondria, and microsome of mice [8]. Flavonoid extracts from *Rhodomyrtus tomentosa* Hassk berries possess excellent radical scavenging activities, and have the ability of increasing serum superoxide dismutase (SOD) and glutathione peroxidase (GSH-Px) levels, and decreasing serum malondialdehyde (MDA) content in normal mice [9]. Flavonoids from *Anoectochilus roxburghii* may ameliorate d-galactose-induced aging in mice by reducing the levels of monoamine oxidase (MAO) and MDA, and enhancing GSH-Px and SOD enzyme activities [10]. A *Livistona chinensis* fruit flavonoids extract alleviates acute liver injury induced by lipopolysaccharides (LPS)/d-galactosamine (D-GalN) by reducing oxidative stress [11]. By increasing GSH-Px, catalase (CAT), and SOD levels, *Ziziphora clinopodioides* flavonoids exert protective effects in human umbilical vein endothelial cells (HUVECs) treated with H_2_O_2_ [12]. Structural diversity has been postulated to be an important element affecting the antioxidant activities of flavonoids. Some studies have compared the antioxidant activities of flavonoid subclasses with different structures [13,14,15,16,17]: (1) The position and number of hydroxyl groups greatly affect the antioxidant activities of flavonoids. (2) The antioxidant activities of flavonoids are dependent on the degree of polymerization. (3) The combination of a 4-carbonyl group and a C2=C3 double bond affects the antioxidant activities of flavonoids. (4) Glycosylate attenuates the antioxidant activities of flavonoids. To our knowledge, the relationships between the antioxidant activities and structures of flavonoids are investigated mainly based on the theoretical inference and chemical analyses, and little information is available on the relationships between flavonoid structures and in vivo antioxidant activities.

**Figure 1:**
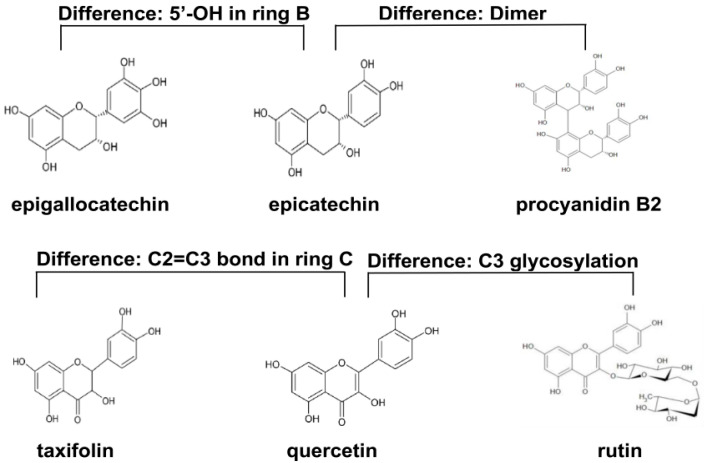
The chemical structures of procyanidin B2, epicatechin, epigallocatechin, taxifolin, quercetin, and rutin.

In the current study, the procyanidin B2, epicatechin, epigallocatechin, taxifolin, quercetin, and rutin were selected. These six flavonoids possessed similar chemical structures, and the differences in the chemical structures were displayed in Figure 1. Moreover, the antioxidant activities of these flavonoids have been reported by some researchers (Table 1). The objective of this study was to compare in vitro and in vivo antioxidant activities of these six flavonoids with similar structures. The in vitro antioxidant activities of six different flavonoids were investigated using the 2,2′-azino-bis-(3-ethylbenzothiazoline-6-sulfonic acid) (ABTS^+^) and 2,2-diphenyl-1-picrylhydrazyl (DPPH•) methods. The attenuated effects of six different flavonoids on oxidative stress and oxidative stress-related inflammation were explored in aging mice treated with D-galactose. Although there are some researches focused on the in vitro and in vivo antioxidant activities of flavonoids [8,9], they use a mixture of flavonoids, which makes it difficult to compare the antioxidant activities of flavonoids with similar structures. Our research compared the in vivo antioxidant activities of flavonoids with similar structures using the model of d-galactose-induced aging mice for the first time.

## 2. Materials and Methods

### 2.1. Chemicals

Epigallocatechin (purity, 98%), epicatechin (purity, 98%), procyanidin B2 (purity, 98%), quercetin (purity, 98%), taxifolin (purity, 98%), and rutin (purity, 98%) were obtained from Jinheng Chemical Co., Ltd. (Xi’an, China). D-galactose was supplied by Sinopharm Chemical Reagent Co., Ltd. (Shanghai, China). DPPH• was supplied by Tixiai Chemical Industrial Development Co., Ltd. (Shanghai, China). Vitamin C and ABTS^+^ were purchased from Shenggong Bioengineering Co., Ltd. (Shanghai, China). The other reagents were provided by Kelong Chemical Reagent Factory (Chengdu, China).

### 2.2. In Vitro Antioxidant Activity Assays 

#### 2.2.1. DPPH• Radical Scavenging Assay

We consulted the original method [24], and made some necessary changes. A 3.9 mL aliquot of 0.063 mM DPPH• solution was mixed with absolute ethanol and 0.1 mL of the diluted sample. The solution was placed in a dark environment for 30 min at room temperature. Then, 200 μL of the final reaction solution was used to determine absorbance at 517 nm in a 96-well plate. The absorbance of DPPH• plus the sample solution was recorded as *A*_1_, the absorbance of ethanol plus the sample solution was recorded as *A*_2_, and the absorbance of the DPPH• plus an 80% methanol solution was recorded as *A*_3_. The DPPH• radical scavenging rate was calculated according to the equation:

$$\mathrm{DPPH}•\mathrm{scavenging}\;\mathrm{rate}\;\left(\%\right)=\frac{A_{3}-\left(A_{1}-A_{2}\right)}{A_{3}}\times 100$$

#### 2.2.2. ABTS^+^ Radical Scavenging Assay

ABTS^+^ radical scavenging was determined as described previously [24], with minor modifications. A 7 mM ABTS radical solution was mixed with a 140 mM K_2_S_2_O_8_ solution to prepare the ABTS solution, which was reacted for 12 h in the dark at room temperature. The solution was diluted with absolute ethanol until the absorbance at 734 nm was 0.700 ± 0.02, and then it was used as the ABTS^+^ reagent. A 5 mL aliquot of ABTS^+^ reagent and 0.2 mL of sample solution were mixed for 6 min at room temperature. The absorbance of the final reacting solution was measured at 734 nm in a 96-well plate. The absorbance of the ABTS^+^ reagent plus the sample solution was recorded as *A*_1_, the absorbance of absolute ethanol plus the sample solution was recorded as *A*_2_, and the absorbance of the ABTS^+^ reagent plus an 80% methanol solution was recorded as *A*_3_. The scavenging rate of the ABTS^+^ radical was calculated according to the equation:

$$ABTS^{+}\;\mathrm{scavenging}\;\mathrm{rate}\;\left(\%\right)=\frac{A_{3}-\left(A_{1}-A_{2}\right)}{A_{3}}\times 100$$

### 2.3. Animal Experiments 

All mice were supplied by the Experimental Animal Center of Chongqing Medical University (Chongqing, China). Under a 12 h:12 h light-dark cycle, Kunming (KM) mice were housed five per cage and fed for 7 days at 22 ± 1 °C. Seventy-two mice (6-week-old, half male and half female) were distributed into nine groups: mice in the D-galactose and control groups were fed normal saline, while mice in the other seven groups was given vitamin C, epicatechin, epigallocatechin, procyanidin B2, quercetin, taxifolin and rutin at a dose of 0.3 mmol/kg·body weight (bw), respectively. All the tested flavonoids were dissolved in 10% aqueous solution of Tween 80. The normal saline, flavonoids and vitamin C were gavaged orally once daily for 8 weeks. Beginning on week 3 of administration, D-galactose (125 mg/kg·bw) was intraperitoneally injected into all the mice once daily for 6 consecutive weeks, except for the control mice. All mice were fasted overnight after 8 weeks, and blood was collected by penetrating the retro-orbital sinus in mice with a sterile hematocrit capillary tube and centrifuged (3000 rpm, 4 °C, 10 min) to obtain the serum. The liver was quickly excised for further analysis. All animal experimental protocols were approved by the Animal Ethics and Experimental Committee of Chongqing Collaborative Innovation Centre for Functional Food (201708005B).

### 2.4. Measurement of Oxidative Stress Indicators and Inflammatory Cytokines 

Serum total antioxidative capacity (T-AOC), SOD, CAT, and GSH-Px levels, as well as nitric oxide (NO) and MDA contents were measured using test kits according to the manufacturer’s instructions (Solarbio, Beijing, China). Serum interleukin (IL)-1β, IL-6, IL-10, and tumor necrosis factor (TNF)-α levels were determined using commercial kits (Solarbio, Beijing, China).

### 2.5. Real-Time Quantitative Polymerase Chain Reaction (RT-PCR) Analysis 

Total RNA was isolated from liver tissues using TRIzol reagent (Tiangen Biotech, Beijing, China). One μg of total RNA was used to synthesize cDNA with the FastQuant cDNA First Chain Synthesis Kit (Tiangen Biotech, Beijing, China). The quantitative RT-PCR reaction volume was 20 μL, which included 1 μL template DNA, 10 μL SYBR^®^ Premix Ex Taq™ (Takara, Otsu, Shiga, Japan), 7 μL ddH_2_O, and 1 μL of each forward and reverse primer. The reaction was initiated with the following conditions: 95 °C for 30 s (preincubation), 40 cycles of 95 °C for 5 s, and 60 °C for 30 s. Glyceraldehyde-3-phosphate dehydrogenase (GAPDH) was used as the internal control gene. The relative mRNA expression was calculated using the 2^−ΔΔCT^ method. Table 2 shows the sequences of primers used in the current study.

### 2.6. Statistical Analysis

Experimental data are presented as mean ± standard deviation. The statistical analysis were performed by one-way analysis of variance followed by Least Significant Difference (LSD) test (SPSS 17.0 software, SPSS Inc., Chicago, IL, USA). A *p*-value < 0.05 was considered significant.

## 3. Results

### 3.1. Effects of Six Flavonoids on In Vitro Antioxidant Activities

**Figure 2:**
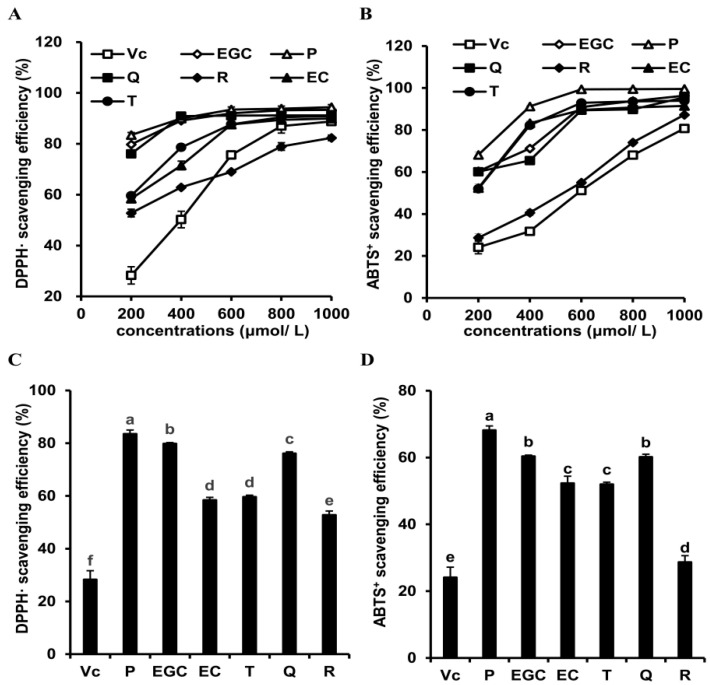
The in vitro antioxidant activities of the six flavonoids. (**A**) The 2,2-diphenyl-1-picrylhydrazyl (DPPH•) scavenging activities of the six flavonoids at different concentrations; (**B**) The 2,2′-azino-bis-(3-ethylbenzothiazoline-6-sulfonic acid) (ABTS^+^) scavenging activities of the six flavonoids at different concentrations; (**C**) The DPPH• scavenging activities of the six flavonoids at a concentration of 200 μmol/L; (**D**) The ABTS^+^ scavenging activities of the six flavonoids at a concentration of 200 μmol/L. Vc, Vit. C group; EC, epicatechin group; EGC, epigallocatechin group; P, procyanidin B2 group; Q, quercetin group; T, taxifolin group; R, rutin group. Results are expressed as means ± SD. Values with different letters are significantly different (*p* < 0.05).

The in vitro antioxidant activities of the six flavonoids were estimated by measuring the clearance rates of DPPH• and ABTS^+^ free radicals. Figure 2A,B shows that the scavenging ability of the six flavonoids and Vitamin C for DPPH• and ABTS^+^ free radicals gradually increased with the increase in the tested concentration. For the current in vitro tests, the higher flavonoids and Vitamin C concentration, the stronger DPPH• and ABTS^+^ scavenging activities. Thus, to clearly compare the differences of the six flavonoids and Vitamin C in the DPPH• and ABTS^+^ scavenging activities, we chose the results obtained at a concentration of 200 μmol/L for further analysis (Figure 2C,D). The DPPH• and ABTS^+^ scavenging activities of Vitamin C were the lowest, compared with those of the six flavonoids (*p* < 0.05). We found that the orders of ability to scavenge DPPH• radicals of the tested flavonoids were procyanidin B2 > epigallocatechin > quercetin > epicatechin ~ taxifolin > rutin. A similar trend for ABTS+ scavenging activity was obtained, procyanidin B2 > epigallocatechin ~ quercetin > epicatechin ~ taxifolin > rutin. These results indicate that the in vitro antioxidant activities of flavonoids were associated with their chemical structures.

### 3.2. Effect of Six Flavonoids on Serum Oxidative Stress Indicators In d-Galactose-Treated Mice

**Figure 3:**
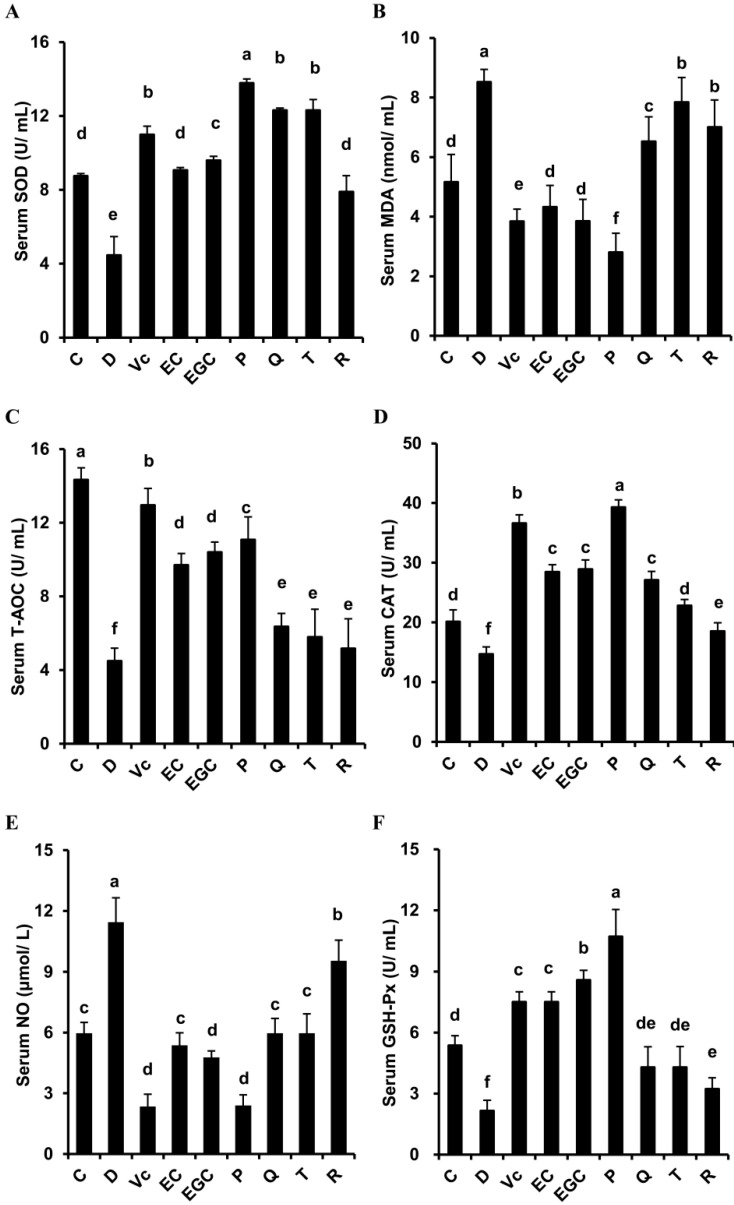
Effect of six flavonoids on serum oxidative stress indicators in d-galactose-treated mice. (**A**) serum superoxide dismutase (SOD); (**B**) serum malondialdehyde (MDA); (**C**) serum total antioxidative capacity (T-AOC); (**D**) serum catalase (CAT); (**E**) serum nitric oxide (NO); (**F**) serum glutathione peroxidase (GSH-Px). C: control group; Vc: Vit. C group; D: d-galactose group; EC: epicatechin group; EGC: epigallocatechin group; P: procyanidin B2 group; Q: quercetin group; T: taxifolin group; R: rutin group. The results are expressed as means ± SD. Values with different letters are significantly different (*p* < 0.05).

As shown in Figure 3, compared with the control mice, serum GSH-Px, CAT, T-AOC, and SOD activities markedly decreased in the D-galactose-treated mice, while d-galactose treatment increased the levels of serum MDA and NO (*p* < 0.05). However, these changes were dissimilarly reversed by the six flavonoids (*p* < 0.05). In comparison to the epicatechin-treated mice, epigallocatechin significantly increased serum GSH-Px and SOD levels (*p* < 0.05), but decreased serum NO contents (*p* < 0.05). The procyanidin B2-treated mice had higher serum GSH-Px, CAT, T-AOC, and SOD activities than those in the epicatechin-treated mice (*p* < 0.05). The MDA and NO levels in the procyanidin B2-treated mice decreased significantly compared with those in the epicatechin-treated mice (*p* < 0.05). Supplementing the mice with quercetin significantly increased serum CAT activity compared with that in the taxifolin-treated mice (*p* < 0.05) but serum MDA levels decreased (*p* < 0.05). SOD and CAT activities increased in the quercetin-treated mice compared to the rutin-treated mice, and NO and MDA concentrations in the quercetin-treated mice decreased (*p* < 0.05). These data reveal that the six flavonoids have different resistance to oxidative stress in D-galactose mice, in the following order: procyanidin B2 > epicatechin, epigallocatechin > epicatechin, quercetin > taxifolin, quercetin > rutin.

### 3.3. Effect of Six Flavonoids on Hepatic Oxidative Stress in d-Galactose-Treated Mice

**Figure 4:**
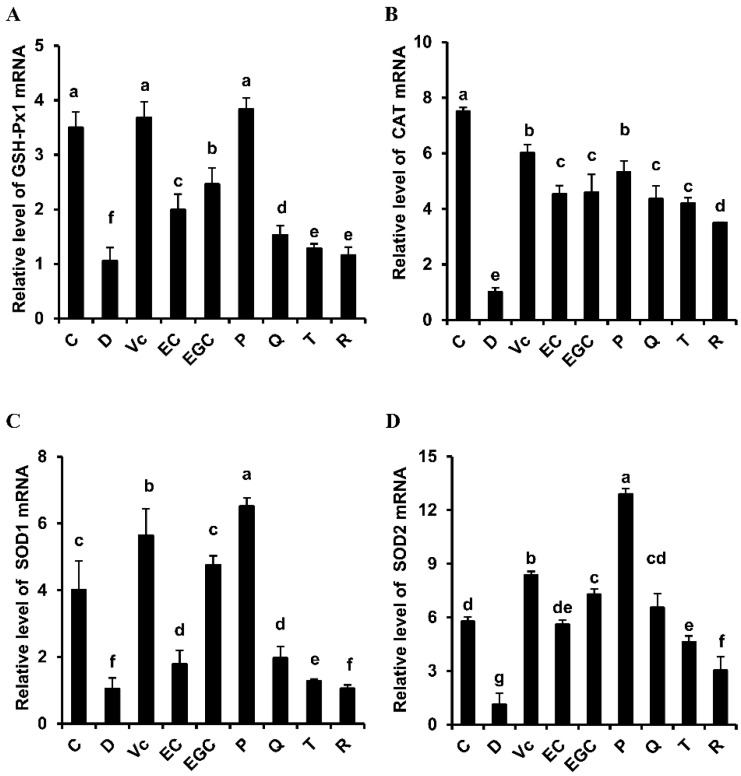
Effect of six flavonoids on hepatic oxidative stress in d-galactose-treated mice. (**A**) glutathione peroxidase 1 (GSH-Px1) mRNA expression level; (**B**) catalase (CAT) mRNA expression level; (**C**) superoxide dismutase (SOD)1 mRNA expression level; (**D**) SOD2 mRNA expression level. C: control group; Vc: Vit. C group; D: D-galactose group; EC: epicatechin group; EGC: epigallocatechin group; P: procyanidin B2 group; Q: quercetin group; T: taxifolin group; R: rutin group. The results are expressed as means ± SD. Values with different letters are significantly different (*p* < 0.05).

Figure 4 shows that hepatic SOD1, SOD2, CAT, and GSH-Px1 mRNA levels decreased in d-galactose-treated mice compared with those in the control mice (*p* < 0.05). However, the flavonoid supplements differentially increased the mRNA levels of SOD1, SOD2, CAT, and GSH-Px1 in the liver tissues of d-galactose-treated mice. SOD1, SOD2, and GSH-Px1 mRNA expression levels increased in the epigallocatechin-treated mice compared with those in the epicatechin-treated mice (*p* < 0.05). SOD1, CAT, SOD2, and GSH-Px1 mRNA expression levels in the epicatechin-treated mice decreased significantly compared with those in the procyanidin-treated B2 mice (*p* < 0.05). SOD1, SOD2, and GSH-Px1 mRNA expression levels were lower in the taxifolin-treated mice than those in the quercetin-treated mice (*p* < 0.05). No difference in CAT mRNA expression was observed between the quercetin and taxifolin-treated mice (*p* > 0.05). The quercetin-treated mice exhibited significantly higher SOD1, CAT, SOD2, and GSH-Px1 mRNA levels than those in the rutin-treated mice (*p* < 0.05). Our data demonstrate that the six flavonoids differentially attenuated hepatic oxidative stress, and the degree of reduction was procyanidin B2 > epicatechin, epigallocatechin > epicatechin, quercetin > taxifolin, quercetin > rutin.

### 3.4. Effect of Six Flavonoids on Oxidative Stress-Related Inflammatory Response in D-Galactose-Treated Mice

**Figure 5:**
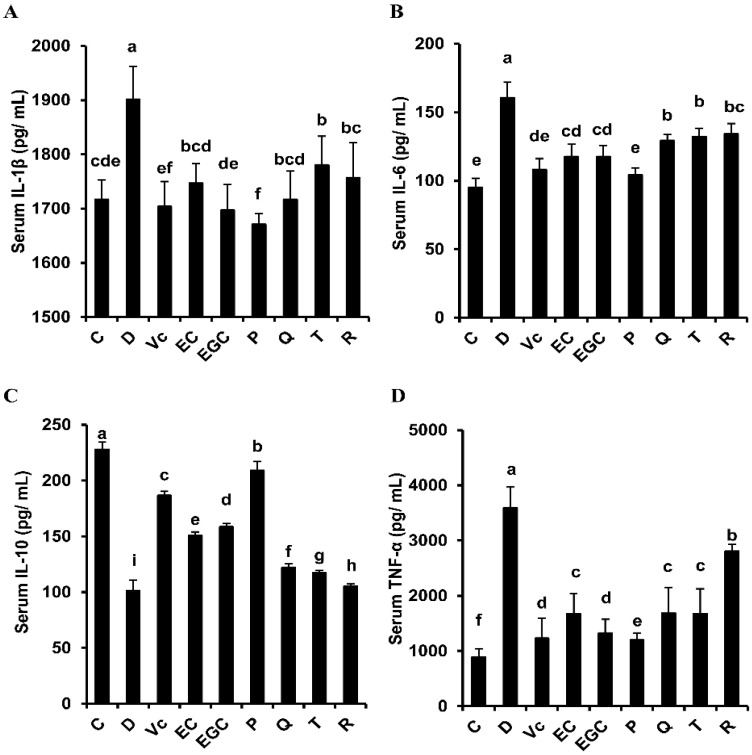
Effect of six flavonoids on oxidative stress-related inflammatory response in d-galactose-treated mice. (**A**) interleukin (IL)-1β; (**B**) IL-6; (**C**) IL-10; (**D**) tumor necrosis factor (TNF)-α. C: control group; Vc: Vit. C group; D: D-galactose group; EC: epicatechin group; EGC: epigallocatechin group; P: procyanidin B2 group; Q: quercetin group; T: taxifolin group; R: rutin group. The results are expressed as means ± SD. Values with different letters are significantly different (*p* < 0.05).

As shown in Figure 5, d-galactose treatment significantly increased the levels of serum inflammatory cytokines, including IL-1β, IL-6, IL-10 and TNF-α (*p* < 0.05). However, these increases were differentially reduced by the flavonoid supplements. Epigallocatechin significantly increased anti-inflammatory factor IL-10 level and decreased TNF-α level compared to epicatechin (*p* < 0.05). Lower levels of TNF-α, IL-1β, and IL-6 and higher level of IL-10 were detected in the procyanidin B2-treated mice compared with the epicatechin-treated mice (*p* < 0.05). IL-10 content was higher in the quercetin-treated mice than that in the taxifolin-treated mice (*p* < 0.05). TNF-α, IL-1β, and IL-6 levels were similar in the taxifolin-treated and quercetin-treated mice (*p* > 0.05). IL-6 and IL-1β levels were similar in the rutin-treated and quercetin-treated mice (*p* > 0.05), but IL-10 level was significantly higher in the quercetin-treated mice than that in the rutin-treated mice (*p* < 0.05). The TNF-α level in the quercetin-treated mice was significantly lower than that recorded in the rutin-treated mice (*p* < 0.05). These results show that the six flavonoids differentially reduced serum inflammatory cytokine levels in the following order: procyanidin B2 > epicatechin, epigallocatechin > epicatechin, quercetin > taxifolin, quercetin > rutin.

## 4. Discussion

The DPPH• and ABTS^+^ assays are widely used to evaluate the in vitro antioxidant activities of flavonoids [25,26]. These two methods are based on the single electron transfer mechanism and involve reduction of a colored oxidant. To be more specific, when in the presence of the antioxidant compounds with the ability of transferring an electron or donating hydrogen, the purple DPPH• is reduced to colorless 1,1-diphenyl-2-picryl hydrazine, whereas the blue-green ABTS radical is reduced to its colorless neutral form [27]. The color of these solutions is lost with the increased concentration of antioxidant compounds, which may be monitored spectrophotometrically. The type of model solution is reported to affect the reaction of flavonoids with DPPH•. The DPPH• scavenging activity of flavonoids can be better assayed in the polar protic solutions, such as methanol, ethanol, and water, since these hydrogen bond donating solvents contribute to the process of single-step hydrogen atom transfer and sequential proton loss electron transfer [28]. Because the in vitro antioxidant activities of flavonoids in aqueous solutions are susceptible to some factors, such as temperature and pH [29,30,31], we used methanol as the solvent of flavonoids and performed the in vitro antioxidant activity assay at room temperature. In the current study, our results showed that the procyanidin B2 had the strongest in vitro free radical scavenging capacity, but rutin possessed the lowest scavenging activity. The free radical scavenging capacities of the six flavonoids were summarized in Table 3. The epigallocatechin was found to possess higher DPPH• and ABTS^+^ radical scavenging activities, compared with epicatechin. The high in vitro antioxidant activity of epigallocatechin is considered to be associated with the number of its phenolic hydroxyl groups, since the B ring hydroxyl can donate hydrogen and an electron to radical species, and make radical species to be stabilized [17,32]. The scavenging activities of quercetin on DPPH• and ABTS^+^ free radicals was higher than those of rutin. It is reported that the presence of sugar moiety on the basic ring skeleton attenuates the in vitro antioxidant activity of rutin, and this sugar substitution at three positions on the C ring is a major detrimental factor of the antioxidant activities of flavonoids [33]. Moreover, compared with quercetin, rutin has a larger structure, which makes it diffuse less readily in the model solution, thereby leading the reduced free radical scavenging ability [34]. In comparison with taxifolin, the structure of quercetin has a C2=C3 double bond conjugated with the aromatic ring, which causes the hydroxyl group linked to the carbon 3 to easily undergo monoelectronic oxidation, thereby showing stronger antioxidant activity [35]. The procyanidin B2 possesses a higher ability to scavenge the DPPH• and ABTS^+^ radicals than epicatechin. One reason is that the amount of phenolic OH group in the procyanidins B2 structure is more than that of epicatechin; the other reason is that the hydrogen at C2 in the dimer is easily abstracted by DPPH•, which makes the benzylic C2 position another reaction site with free radicals [36].

D-galactose causes premature aging in mice, which is similar to the natural aging process [37]. Oxidative stress plays a key role in various pathologies and is closely related to the aging process. The damage caused by oxidative stress can be resisted by the enzymatic antioxidant defense system [38,39]. The activities of antioxidant defense enzymes (GSH-Px, CAT, and SOD) in the liver tissues of D-galactose-treated mice are low [40]. A decrease in SOD activity and an increase in MDA content are found in aging mice, and are commonly considered markers of oxidative stress [41]. T-AOC activity is associated with the non-enzymatic antioxidant defense system, and decreased levels of T-AOC are found in the liver and stomach tissues of D-galactose-treated mice [42]. NO is a free radical gas molecule linked to cell damage effects [43], and MDA and NO levels increase in D-galactose-treated mice, but are attenuated by tetrahydropalmatine [44]. The six flavonoids tested here reversed serum SOD, GSH-Px, CAT, and T-AOC levels inhibited by D-galactose, and serum MDA and NO levels were suppressed by the six flavonoids. Liver SOD1, CAT, SOD2, and GSH-Px1 mRNA expression levels decreased in the D-galactose-treated mice, while the six flavonoids enhanced the expression levels of these genes. These results suggest that protection of the cells against D-galactose-induced oxidative stress by the six flavonoids was related to the regulation of antioxidant enzymes, lipid peroxidation, and the expression of liver antioxidant genes. Oxidative stress is thought to play a key role in promoting the secretion of inflammatory cytokines and gene expression of proinflammatory cytokines [45,46]. IL-10 represses the proinflammatory response and serum IL-10 decreases significantly in the D-galactose-treated mice [7,47]. IL-6 exacerbates the risk of age-related diseases, which may be related to chronic stress [48]. Serum TNF-α, IL-6, and IL-1β levels increase in D-galactose-induced aging mice [49]. Diosgenin significantly decreases liver IL-1β, TNF-α, and IL-6 levels compared with those in the LPS/D-GalN-treated mice [50]. Flavonoids from *Lotus plumule* relieve the inflammatory response by inhibiting TNF-α, IL-6, and IL-1β levels [51]. Flavonoids reduce the inflammatory response by reducing IL-6, TNF-α, and IL-1β expression [52]. In the current study, serum TNF-α, IL-1β, and IL-6 levels increased significantly in the d-galactose-treated mice, which were reversed by the six flavonoids. Moreover, the six flavonoids promoted IL-10 secretion to varying degrees. Taken together, the six flavonoids differentially alleviated d-galactose-induced oxidative stress and oxidative stress-related inflammation in mice.

In our study, the different efficiencies of the six flavonoids on all the in vivo and in vitro tests were summarized in Table 3. Table 3 shows that procyanidin B2 had higher antioxidant capacity compared to epicatechin, and epigallocatechin had much stronger antioxidant capacity than that of epicatechin. We also found that quercetin had adequate antioxidant capacity, which was clearly superior to that of taxifolin and rutin. The different antioxidant efficiencies of flavonoids are involved with their chemical structures. There is no 5′-OH group in the B ring in epicatechin in comparison to epigallocatechin. The amount of OH has a vital effect on the antioxidant activities of flavonoids, and is positively correlated with the antioxidant activities of flavonoids [53]. After oral administration of epigallocatechin and epicatechin in rats, the level of epigallocatechin in the plasma is significantly higher than that of epicatechin, which indicates epigallocatechin has higher oral bioavailability in comparison to epicatechin [54]. A C ring with a C2=C3 double bond is the major structural difference between quercetin and taxifolin. An experiment in rats shows that the bioavailability of quercetin (4.11%) after oral administration is much higher than that of oral administration of taxifolin (0.49%) [55,56]. Furthermore, lactase–phlorizin hydrolase can effectively hydrolyze quercetin and produce easily absorbed aglycone in the intestinal lumen, however, rutin cannot be hydrolyzed by intestinal β-glucosidases, and is only metabolized by microorganisms in the cecum and colon [57]. The single difference in the structures of quercetin and rutin is the presence/absence of glycosylate in the ring. ROS production is considered to be associated with protein kinase C (PKC) isoenzyme. ROS can activate PKC signaling independently of Ca^2+^, but it in turn promotes the ROS production, thus forming a vicious circle [58,59]. It is reported that quercetin can effectively inhibit PKC activity, while rutin significantly increases PKC activity [60]. Procyanidin B2 exists as a dimer, which is lacking in epicatechin. Compared with epicatechin, procyanidin B2 can reduce the loss of other antioxidant nutrients and decrease the formation of oxidation products, which contributes to the elevation of the total antioxidant capacity in vivo, according to Chinese Dietary Reference Intakes (2013).

It has been reported that the antioxidant efficacies of many flavonoids differ between in vitro and in vivo results. Procyanidins-rich apple extracts show high antioxidant capacity in vitro, while the plasma, after healthy volunteers eat five apples, fails to protect lipids from oxidation. This inconsistence is attributed to the poor absorption and metabolic conversion of apple polyphenols [61]. Although chrysin shows many health-promoting effects including antioxidant and anti-inflammatory activities in in vitro studies, its exceedingly low absorption after oral administration makes its therapeutic application to be significantly limited [62]. Therefore, the bioavailability of flavonoids plays a vital role for their bioactivities in vivo. The chemical structures of flavonoids and the interaction with gut microflora are associated with the bioavailability of flavonoids [63]. In our study, although there was a little bit different between in vitro and in vivo results in terms of some specific indexes, the results obtained from in vitro and in vivo tests were consistent on the whole. The chemical structures of flavonoids have significant impacts on their in vitro and in vivo antioxidant activities. However, how the in vivo bioavailability of these flavonoids affects the structure–activity relationship need to be further investigated.

## 5. Conclusions

Based on the in vitro and in vivo results, the order of antioxidant activities of the six flavonoids was P > EC, EGC > EC, Q > T, Q > R. The differences in the antioxidant capacities of these flavonoids were associated with their chemical structures. The antioxidant capacities of flavonoids were enhanced by pyrogallol, dimerization, and a C2=C3 double bond; C3 glycosylation significantly reduced the antioxidant activities of flavonoids. Our study provides important data for the structure–antioxidant relationships of flavonoids in vivo.

## Figures and Tables

**Table 1:** The antioxidant activities of procyanidin B2, epicatechin, epigallocatechin, taxifolin, quercetin, and rutin.

Flavonoid	The Antioxidant Activities
Procyanidin B2	Procyanidin B2, a widely distributed dimer in natural procyanidins, protects colonic cells against oxidative stress-induced injury [18].
Epicatechin	Epicatechin can exert anti-inflammatory and antioxidant effects, and it can pass through the blood brain barrier to provide neuroprotection [19].
Epigallocatechin	Two most important antioxidants in tea are epigallocatechin gallate and epigallocatechin, and the A ring of epigallocatechin has been demonstrated to be an antioxidant site [20].
Taxifolin	Taxifolin is a free radical scavenger, and its antioxidant capacity is superior to ordinary flavonoids [21].
Quercetin	Quercetin, a ubiquitously distributed flavonoid in plants, has a potent free radical scavenging capacity [22].
Rutin	The antioxidant activity is considered as one of the important pharmacological effects of rutin [23].

**Table 2:** The sequences of primers used in the current study.

Primer Name	Accession Number	Forward Primer (5′ to 3′)	Reverse Primer (5′ to 3′)
GAPDH	NM_001289726.1	AGGTCGGTGTGAACGGATTTG	GGGGTCGTTGATGGCAACA
SOD1	NM_011434.2	AACCAGTTGTGTTGTCAGGAC	CCACCATGTTTCTTAGAGTGAGG
SOD2	NM_013671.3	CAGACCTGCCTTACGACTATGG	CTCGGTGGCGTTGAGATTGTT
CAT	NM_009804.2	GGAGGCGGGAACCCAATAG	GTGTGCCATCTCGTCAGTGAA
GSH-Px1	NM_008160.6	CCACCGTGTATGCCTTCTCC	AGAGAGACGCGACATTCTCAAT

**Table 3:** The different efficiencies of the six flavonoids on all the in vivo and in vitro tests. EC, epicatechin group; EGC, epigallocatechin group; P, procyanidin B2 group; Q, quercetin group; T, taxifolin group; R, rutin group. The symbol “>” means the level of the former is higher than the latter in the corresponding test; The symbol “<” means the level of the former is less than the latter in the corresponding test; The symbol “≈” means there is no significant difference between the former and the latter in the corresponding test; The symbol “+“means the corresponding structure exists in flavonoid; The symbol “-” means the corresponding flavonoid is absent in flavonoid.

Index	Dimer	5′-OH in Ring B	C2=C3 Bond in Ring C	C3 Glycosylation
DPPH• scavenging efficiency	P (+) > EC (-)	EGC (+) > EC (-)	Q (+) > T (-)	Q (-) > R (+)
ABTS^+^ scavenging efficiency	P (+) > EC (-)	EGC (+) > EC (-)	Q (+) > T (-)	Q (-) > R (+)
Serum SOD	P (+) > EC (-)	EGC (+) > EC (-)	Q (+) ≈ T (-)	Q (-) > R (+)
Serum T-AOC	P (+) > EC (-)	EGC (+) ≈ EC (-)	Q (+) ≈ T (-)	Q (-) ≈ R (+)
Serum CAT	P (+) > EC (-)	EGC (+) ≈ EC (-)	Q (+) > T (-)	Q (-) > R (+)
Serum GSH-Px	P (+) > EC (-)	EGC (+) > EC (-)	Q (+) ≈ T (-)	Q (-) ≈ R (+)
Serum NO	P (+) < EC (-)	EGC (+) < EC (-)	Q (+) ≈ T (-)	Q (-) < R (+)
Serum MDA	P (+) < EC (-)	EGC (+) ≈ EC (-)	Q (+) < T (-)	Q (-) < R (+)
Hepatic GSH-Px1 mRNA	P (+) > EC (-)	EGC (+) > EC (-)	Q (+) > T (-)	Q (-) > R (+)
Hepatic CAT mRNA	P (+) > EC (-)	EGC (+) ≈ EC (-)	Q (+) ≈ T (-)	Q (-) > R (+)
Hepatic SOD1 mRNA	P (+) > EC (-)	EGC (+) > EC (-)	Q (+) > T (-)	Q (-) > R (+)
Hepatic SOD2 mRNA	P (+) > EC (-)	EGC (+) > EC (-)	Q (+) > T (-)	Q (-) > R (+)
Serum IL-10	P (+) > EC (-)	EGC (+) > EC (-)	Q (+) > T (-)	Q (-) > R (+)
Serum IL-1β	P (+) < EC (-)	EGC (+) ≈ EC (-)	Q (+) ≈ T (-)	Q (-) ≈ R (+)
Serum IL-6	P (+) < EC (-)	EGC (+) ≈ EC (-)	Q (+) ≈ T (-)	Q (-) ≈ R (+)
Serum TNF-α	P (+) < EC (-)	EGC (+) < EC (-)	Q (+) ≈ T (-)	Q (-) < R (+)
